# HMGB1 Inhibition During Zymosan-Induced Inflammation: The Potential Therapeutic Action of Riboflavin

**DOI:** 10.1007/s00005-015-0366-6

**Published:** 2015-10-07

**Authors:** Agnieszka Irena Mazur-Bialy, Ewa Pocheć

**Affiliations:** Department of Ergonomics and Exercise Physiology, Faculty of Health Science, Jagiellonian University Medical College, Grzegorzecka 20, 31-531 Kraków, Poland; Department of Glycoconjugate Biochemistry, Institute of Zoology, Jagiellonian University, Kraków, Poland

**Keywords:** Macrophages, Vitamin B2, HMGB1, Inflammation, Sepsis, Peritonitis

## Abstract

Sepsis, also known as systemic inflammatory response syndrome, is a life-threatening condition caused by a pathogenic agent and leading to multiple organ dysfunction syndrome. One of the factors responsible for the excessive intensification of the inflammatory response in the course of inflammation is high-mobility group protein B1 (HMGB1). HMG-1 is a nuclear protein which, after being released to the intercellular space, has a highly pro-inflammatory effect and acts as a late mediator of lethal damage. The purpose of this study was to examine whether the anti-inflammatory action of riboflavin is accompanied by inhibition of HMGB1 release during peritoneal inflammation and zymosan stimulation of macrophages. Peritonitis was induced in male BALB/c and C57BL/6J mice via intraperitoneal injection of zymosan (40 mg/kg). RAW 264.7 macrophages were activated with zymosan (250 µg/ml). Riboflavin (mice, 50 mg/kg; RAW 264.7, 25 µg/ml) was administered 30 min before zymosan, simultaneously with, or 2, 4, 6 h after zymosan. Additionally, mRNA expression of HMGB1 and its intracellular and serum levels were evaluated. The research showed that riboflavin significantly reduces both the expression and the release of HMGB1; however, the effect of riboflavin was time-dependent. The greatest efficacy was found when riboflavin was given 30 min prior to zymosan, and also 2 and 4 h (C57BL/6J; RAW 264.7) or 4 and 6 h (BALB/c) after zymosan. Research showed that riboflavin influences the level of HMGB1 released in the course of inflammation; however, further study is necessary to determine its mechanisms of action.

## Introduction

High-mobility group protein B1 (HMGB1) is a member of the HMG protein family that is common in the nucleus of quiescent cells. Its basic function is connected with DNA binding and nucleosome stabilization, as well as with the regulation of the gene transcription and expression process. Nevertheless, recent studies reported that in certain situations HMGB1 may be released from cells to the intercellular space and, acting via various receptors such as RAGE, TLR2, TLR4 or TLR9, induces a pro-inflammatory activation of neutrophils or macrophages (Huang et al. 2010; Kim et al. 2013). HMGB1 release may occur as a passive leakage from necrotic cells or as an active process therefore of a pro-inflammatory activation of macrophages with endotoxins or pro-inflammatory cytokines (Andersson and Tracey 2011; Youn et al. 2011). Initially, in the active process HMGB1 is transported to cytoplasm, while release from the cell requires its acetylation, phosphorylation or methylation (Huang et al. 2010). HMGB1 is described as a late mediator of lethal systemic inflammation, because its level in serum increases after the principle peak of early pro-inflammatory cytokines, reaching its maximum at about 18–24 h or later. The consequence of HMGB1 influence upon immunocompetent cells is the stimulation of releasing inflammatory agents which results in amplification of the immunological response, due to which acute tissue damage occurs (Huang et al. 2010; Wang et al. 1999; Wu et al. 2012). Both sepsis and severe endotoxemia conditions may result in the development of multiple organ dysfunction syndrome (MODS) which occurs as a consequence of the excessive and protracted activation of the immune system (e.g. HMGB1 release). Research shows that the model of zymosan-induced peritonitis, described as the condition of systemic inflammation, is optimum to test MODS, because mechanisms generated thereby best demonstrate changes in the human body (Volman et al. 2005). We previously showed an anti-inflammatory activity of riboflavin in the model of zymosan-induced peritonitis (Mazur-Bialy et al. 2011, 2012). The purpose of this study was to evaluate the effect of riboflavin administration on HMGB1 gene expression and protein release from cells. The study was conducted in the course of zymosan-induced peritonitis in C57BL/6J and BALB/c mice as well as on zymosan-stimulated macrophages RAW 264.7. Protective effect of riboflavin administration was mentioned before (Al-Harbi et al. 2014; Bütün et al. 2015; Cheung et al. 2014; Mal et al. 2013; Mazur-Bialy et al. 2011; Shih et al. 2010; Toyosawa et al. 2004). Previous studies had shown that riboflavin reduces the inflammatory response in the course of severe endotoxemia, inhibits the release of pro-inflammatory cytokines (Mal et al. 2013; Mazur-Bialy et al. 2011, 2012; Toyosawa et al. 2004) and decreases the mortality rate of animals in septic shock (Shih et al. 2010; Toyosawa et al. 2004) as well as reduces the hepatocellular injury and hepatotoxicity (Al-Harbi et al. 2014; Sanches et al. 2014). Therefore, determining whether the observed anti-inflammatory activity of riboflavin is connected with HMGB1 release seems to be important.

## Materials and Methods

### Animals

The study was conducted on 6-week-old male mice of C57BL/6J and BALB/c strains purchased from a licensed animal colony (Slaboszow, Poland). The animals were kept in standard conditions: temperature 20 ± 2 °C, 12:12 LD, with free access to water and food. All procedures involving the animals were approved by the Local Committee on Animal Care at the Jagiellonian University (License No. 77/2011).

### Experimental Peritonitis

After 1 week of acclimatization, the animals were divided into experimental groups on a random basis (four animals per group). Peritonitis was induced by an intraperitoneal (i.p.) injection of zymosan A (Z, 40 mg/kg; Sigma-Aldrich, St. Louis, MO, USA). Some animals received a riboflavin i.p. injection (50 mg/kg; Sigma-Aldrich, St. Louis, MO, USA) for 30 min either before zymosan, with zymosan or 2, 4, 6 h after i.p. zymosan injection. Control animals received only sterile 0.9 % phosphate buffered saline (PBS; Polfa Kutno, Poland). Zymosan and riboflavin were prepared freshly in sterile PBS. At selected time points of inflammation (0, 2, 6, 24, 30 h), the mice were killed by decapitation. The peritoneal fluid and blood were retrieved. Cell pellets, fluids and serum were stored at −20 °C until further analysis.

### Cell Culture

Macrophage cell line RAW 264.7, free of mycoplasma, was obtained from the European Culture Collection (Sigma-Aldrich, St. Louis, MO, USA). The cells were cultured in DMEM medium (Sigma-Aldrich, St. Louis, MO, USA) supplemented with antibiotics and 10 % fetal bovine serum (Sigma-Aldrich, St. Louis, MO, USA). For the experiments, cells were cultured with or without riboflavin (25 µg/ml) administered 30 min before zymosan (250 µg/ml), with zymosan or 2, 4, 6 h after zymosan stimulation. The cell pellets and supernatants were collected at selected time points during the experiments (0, 18, 24, 30 h) and were stored store at −20 °C until further analysis.

### HMGB1 Expression

Total RNA from RAW 264.7 cells and mice peritoneal leukocytes were isolated using an RNeasy Plus Mini Kit (Qiagen, Hilden, Germany; No. 74134) at hour 24 of the experiment. RNA was reverse transcribed using a High Capacity RNA-to-cDNA Kit (Applied Biosystems, Foster City, CA, USA; No. 4387406). Real-time PCR was carried out in a StepOne Plus system (Applied Biosystems, Foster City, CA, USA) using TaqMan Gene Expression Master Mix and TaqMan Gene Expression Assays (Applied Biosystems, Foster City, CA, USA) for HMGB1 (Assay ID: Mm00849805_gH). PCR amplification was performed under the following conditions: 95 °C denaturation for 10 min, followed by 40 cycles of 95 °C for 15  s, then primer hybridization and extension at 60 °C for 1 min. Glyceraldehyde phosphate dehydrogenase (GAPDH) was used as a reference gene (Applied Biosystems, Foster City, CA, USA; Assay ID: Mm99999915_g1). Four independent replications for each riboflavin concentration were performed. The relative transcript expression was calculated using the $2^{{ - \varDelta \varDelta C_{\text{t}} }}$ method as described by Livak and Schmittgen (2001).

### Intracellular HMGB1 Concentration and Extracellular Release

To assess the level of HMGB1 released by both zymosan-stimulated RAW 264.7 macrophages as well as mice peritoneal leukocytes, a commercial ELISA kit was used (IBL International, Hamburg, Germany). The HMGB1 level was tested in previously frozen cell culture supernatant and mice serum collected at hour 30 of the experiment. The analysis was performed according to the manufacturer’s instructions. Moreover, for evaluation of the intracellular (cytoplasmic and nuclear) HMGB1 content, RAW 264.7 cells were harvested 18 h after stimulation, washed twice in PBS and then the cytoplasmic and nuclear protein was isolated using a commercial Cell Extraction kit (Applied Biosystem; Foster City, CA, USA), according to the manufacturer’s procedure. Before analysis, samples of both cytoplasmic and nuclear proteins were normalized for protein concentrations. The levels of HMGB1 were assessed as described above.

### Quantification of TNF-α and IL-6 Levels

The cytokine levels were measured in the supernatant or peritoneal fluid collected in the second (tumor necrosis factor (TNF)-α) or sixth hour (interleukin (IL)-6) after zymosan administration in samples frozen prior to analysis at −20 °C. The analyses were performed using the commercial ELISA Kit (Diaclone, France; for both) according to the instructions provided by the producer.

### Statistical Analysis

Data were analyzed by the Dunkan’s new multiple range test 3.1. The level of statistical significance was set at 0.05. All data were expressed as mean ± standard deviation (*X* ± SD).

## Results and Discussion

High-mobility group protein B1 is a protein involved in many biological processes. Apart from its role as a nucleus factor, it also contributes in the immune response or pathomechanism of various immune-mediated diseases such as sepsis or rheumatoid arthritis (Huang et al. 2010).

**Fig. 1:**
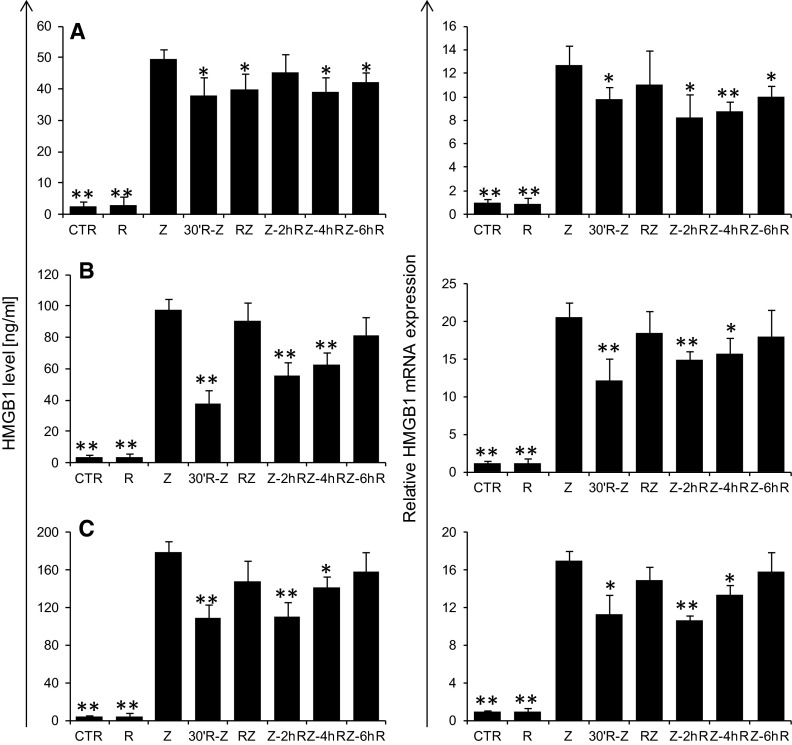
Influence of riboflavin on HMGB1 release (*left panel*) in the 30th hour and expression of HMGB1 mRNA (*right panel*) in the 24th hour of inflammatory process induced by i.p. administration of zymosan (40 mg/kg) at BALB/c (**a**) and C57BL/6J mice (**b**) and by RAW 264.7 macrophages (**c**) stimulated with zymosan (250 µg/ml). The inflammation was modulated by riboflavin supplementation (50 mg/kg for mice or 25 µg/ml for RAW 264.7 macrophages) 30 min prior to zymosan, simultaneously with zymosan or 2, 4, 6 h after zymosan. The results are presented as mean values with a standard deviation. *N* = 4; *p* < 0.05. Statistically significant differences determined relative to control values are marked with the following *symbols* over the *bars*: *p < 0.05; **p < 0.01

This study shows that peritoneal administration of zymosan induces HMGB1 release, which is responsible for significant increase in its serum level in the 30th hour of inflammation. Moreover, an interstrain difference in the quantity of released HMGB1 may also be observed. C57BL/6J mice with predominant Th1-response presented HMGB1 levels nearly 51 % higher than BALB/c mice with predominant Th2-response. As reported by Messmer et al. (2004), HMGB1 induced the Th1 polarization of the immune response that dominates in C57BL/6J mice. It can be argued that this phenomenon may have a relationship with an interstrain difference at the level of maximal HMGB1 release observed between C57BL/6J and BALB/c mice. As expected, administration of riboflavin reduced the level of released HMGB1. The greatest efficacy for the inhibition of HMGB1 release was observed in the case of riboflavin supplementation 30 min before zymosan, where in two strains this level was reduced by 24 % or 61 % in BALB/c mice (Fig. 1a) or C57BL/6J mice (Fig. 1b), respectively, and by 39 % in RAW 264.7 macrophages (Fig. 1c). Simultaneous administration of zymosan and riboflavin significantly reduced HMGB1 levels only in BALC/c mice (21 %; Fig. 1b), while administrating riboflavin 2, 4 or 6 h after the stimulation reduced HMGB1 levels in BALB mice by 21 and 15 % (in the fourth and sixth hour, respectively; Fig. 1b), in C57BL mice by 47 and 34 % (in the second and fourth hour, respectively; Fig. 1b), and in RAW 264.7 macrophages by 38 and 21 % (in the second and fourth hour, respectively; Fig. 1c).

HMGB1 release into the extracellular environment proceeds through either passive or active transport. In passive transport, HMGB1 is released by cells with damaged membrane integrity, as can be observed in the case of necrotic or late apoptotic cells undergoing second necrosis. In such cases, HMGB1 conveys the signal of cell damage to the neighboring structures intensifying inflammatory processes and activating innate and adaptive immune responses (Andersson and Tracey 2011; Huang et al. 2010). Research shows, however, that apoptotic cells keep HMGB1 bound with DNA and do not release it into the extracellular space (Scaffidi et al. 2002). Nevertheless, the excessive accumulation of apoptotic cells in the inflammatory area stimulates macrophages to HMGB1 release (Qin et al. 2006). We did not demonstrate any impact of riboflavin on the intensification of apoptosis and necrosis in the course of peritonitis. Therefore, riboflavin-modulated changes in the level of released HMGB1 are likely to be a result of an active rather than a passive mechanism (data not shown). After activation by an inflammatory agent, HMGB1 is initially translocated from the nucleus to the cell cytoplasm where its level increases up to the 18th hour and is maintained up to 36 h. HMGB1 active release requires its acetylation, phosphorylation or methylation. Only at the 18th hour does the mRNA expression level for HMGB1 increase in the cell nucleus and this is maintained for up to 48 h. Re-formed HMGB1 may then be released into the intercellular space where it intensifies the activation of immunocompetent cells (Wu et al. 2012). In the present study the level of mRNA expression for HMGB1 genes were assessed both in cells isolated from the inflammatory area and in RAW 264.7 macrophages; moreover, intracellular HMGB1 content was measured in RAW 264.7 cell lysates.

Both in peritoneal leukocytes and RAW 264.7 cells, the administration of zymosan induced an elevation in HMGB1 mRNA expression (Fig. 1, right panel). Moreover, our data demonstrated the significant reduction of HMGB1 expression in all groups upon administration of riboflavin 30 min prior to zymosan and in the second and fourth hour of inflammation in C57BL mice (41, 27 and 23 %, respectively; Fig. 1b) and RAW 264.7 cells (33, 37 and 21 %, respectively; Fig. 1c), and additionally, in the sixth hour in BALB mice (27, 35, 31 and 21 %, respectively; Fig. 1a). The analysis of cytoplasmic and nuclear level of HMGB1 at the 18th hour of stimulation, allowing for indirect evaluation of its translocation from the nucleus to the cytoplasm, showed no difference between the study groups. This result may suggest that a reduction in the serum or supernatant HMGB1 level observed after riboflavin administration is not the outcome of blocking its transportation to the cytoplasm. However, it has to be remembered that, in these early hours the mRNA expression is initiated and the whole HMGB1 accumulated in the cell where it is a constitutive component of the nucleus. It seems, therefore, that changes in HMGB1 level observed after riboflavin treatment may be due to reduction in its subsequent expression at the mRNA level or impaired maturation of HMGB1 in the cytoplasm—as described earlier in the text by pointing to the need of its acetylation, phosphorylation or methylation prior to the process of active release from the cell.

**Fig. 2:**
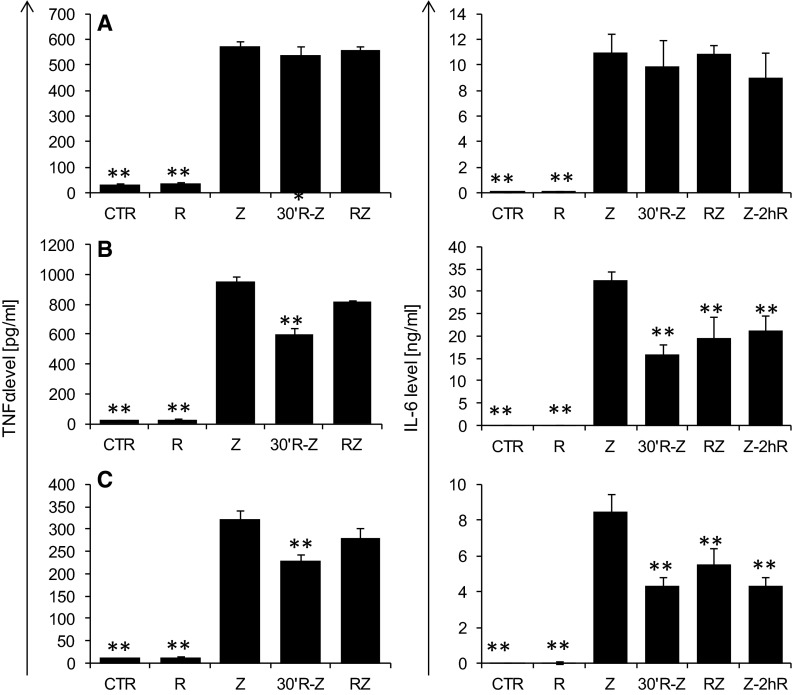
The effect of riboflavin on TNF-α release (*left panel*) in the second hour of inflammation and IL-6 (*right panel*) in the 6th hour of inflammatory process induced by i.p. administration of zymosan (40 mg/kg) at BALB/c (**a**) and C57BL/6J mice (**b**) and by RAW 264.7 macrophages (**c**) stimulated with zymosan (250 µg/ml). The inflammation was modulated by riboflavin supplementation (50 mg/kg for mice or 25 µg/ml for RAW 264.7 macrophages) 30 min prior to zymosan, simultaneously with zymosan or 2, 4 h after zymosan. The results are presented as mean values with a standard deviation. *N* = 4; *p* < 0.05. Statistically significant differences determined relative to control values are marked with the following *symbols* over the *bars*: *p < 0.05; **p < 0.01

It should also be mentioned that HMGB1 release occurs as a result of stimulating cells with both the endotoxin and pro-inflammatory cytokines (Huang et al. 2010; Wang et al. 1999). Assessment of TNF-α and IL-6 levels in C57BL mice and RAW 264.7 macrophages in the second and sixth hour of the inflammatory process demonstrated a significantly lower level of both TNF-α (Fig. 2, left panel) and IL-6 (Fig. 2, right panel) upon riboflavin administration 30 min prior to zymosan; and also that of IL-6 in groups supplemented with riboflavin 2 and 4 h after zymosan. The level of TNF-α was reduced by 37 and 29 %, respectively, in C57BL/6J mice and RAW 264.7 macrophages, while IL-6 was reduced in C57BL/6J and RAW 264.7 by more than 35 % at all tested time points. This observation may suggest that administration of riboflavin has an anti-inflammatory effect, reducing the level of cytokines stimulating cells to HMGB1 release. In the case of BALB mice, no effect of riboflavin administration on TNF-α and IL-6 release was observed, which is in agreement with our previous findings. Considering the above mentioned results, it may be assumed that the inhibition of HMGB1 release may be followed by at least two mechanisms. In case of C57BL/6J and RAW 264.7 macrophages, HMGB1 inhibition was associated with reduction of pro-inflammatory cytokines, which was not observed in BALB/c mice in which HMGB1 inhibition was found. Therefore, there is a need to search for another mechanism of HMGB1 inhibition.

In conclusion, numerous studies showed that riboflavin has both analgesic and anti-inflammatory effects, reducing the mortality rate from septic shock. This study demonstrated for the first time that administration of riboflavin in both zymosan-induced peritonitis and in vitro macrophages model reduced the release of HMGB1—the key agent responsible for multiple organ dysfunctions and lethality in the case of sepsis or severe endotoxemia. However, future studies are needed to determine whether or not the application of riboflavin in other inflammatory models will also result in the impairment of HMGB1 release. Also, it has to be verified if inhibition of the HMGB1 release occurs either as a direct effect of riboflavin administration or as a secondary effect resulted from the inhibition of the key inflammatory cytokine after riboflavin treatment.
